# Assessment of SARS-CoV-2 genome sequence recovery from four lateral flow device products available in the UK

**DOI:** 10.1099/jmm.0.002144

**Published:** 2026-03-23

**Authors:** Kuiama Lewandowski, Matthew Stokes, Alexandra Alexandridou, Abigail Fenwick, Mia L. White, Jack Crook, Karly-Rai Rogers-Broadway, Catherine Ryan, Deborah A. Williamson, Richard Vipond, Steven T. Pullan

**Affiliations:** 1 UK Health Security Agency, Specialised Microbiology and Laboratories, London, UK

**Keywords:** lateral flow device, SARS-CoV-2, sequencing

## Abstract

**Introduction.** Lateral flow tests have played a key role in the response to the COVID-19 pandemic and are likely to be a major component of diagnostic strategies to combat future outbreaks of infectious disease.

**Gap Statement.** One challenge posed by widescale use of lateral flow tests in the community is the loss of sequence information to track virus evolution and epidemiology.

**Aim.** Beyond their primary diagnostic function, it has been demonstrated that recovery of viral RNA from positive lateral flow devices (LFDs) for genome sequencing purposes is possible. We, therefore, aimed to assess the robustness and broader applicability of this process.

**Methodology.** We evaluated SARS-CoV-2 RNA recovery and subsequent sequencing from eluates from the four major LFDs in use in the UK, testing both cultured virus and residual clinical nasal swab samples.

**Results.** Our results demonstrated that sequencing from LFD eluates is possible, at clinically relevant titres, within a reasonable processing time frame post-use, and gave sequences concordant with routine methods. However, results varied across the four devices used.

**Conclusion.** Our results highlight that if sequencing from LFD eluates is to be put into routine use, there is the requirement for refinement of existing or second-generation designs, optimized for sequencing as an intended output from positive tests.

## Introduction

Genomic sequencing of SARS-CoV-2 has had a major role in the public health response to the COVID-19 pandemic, enabling mapping of viral transmission at global and local levels, informing infection control measures and identifying and tracking the emergence of new variants [1].

Viral transport media from nasal/oral swabs have been the most widely utilized sample source for SARS-CoV-2 whole-genome sequencing (WGS) pipelines. Typically, these have been residual material from diagnostic testing, taken forward for genome sequencing (most commonly via whole-genome tiling amplicon-based methods) upon positivity [2]. However, point-of-care lateral flow device (LFD) tests, including self-test kits, are replacing PCR as diagnostics in many settings [3], meaning opportunities for genomic characterization of circulating variants are increasingly limited.

Multiple studies have demonstrated the possibility of retrieving viral nucleic acid from positive LFDs. Martin *et al.* [4] described an approach for WGS of SARS-CoV-2 from positive LFDs and demonstrated the application of this technique to PanBio and InnoScreen devices collected as part of clinical care. The same device brands were also used to demonstrate detection and whole-genome sequencing of non-SARS-CoV-2 respiratory viruses present on the COVID-19 LFD [5]. Macori *et al.* [6] demonstrated that SARS-CoV-2 sequencing was possible from PanBio and Clinitest devices. Rector *et al.* [7] demonstrated that genomic material of SARS-CoV-2, influenza virus, adenovirus and rotavirus can also be retrieved from their corresponding positive LFDs, tested across nine different device brands (Roche, FlowFlex, Newgene, Coris, Alltest, Boson, OrientGene, AMP and Nadal), showing stability for up to 3 months post-use storage at room temperature, but noting that there were significant differences in RNA yields obtained from different device buffers, highlighting the requirement for individual device testing prior to routine use in sequencing.

In this study, we aim to perform an assessment focussed on those LFD brands currently in use in the UK, with a focus on the feasibility of performing the assay in a real-world relevant processing time frame.

## Methods

### LFDs tested

Four brands of SARS-CoV-2 LFD widely used in the UK were procured for testing:

- FlowFlex COVID-19 Antigen Rapid Test (FlowFlex)
- COVID-19 SureScreen Lateral Flow Self-Test (SureScreen)
- OrientGene Rapid Covid-19 (Antigen) Self-Test (OrientGene)
- INNOVA SARS-CoV-2 Rapid Antigen Test (Innova)

### Inactivated SARS-CoV-2

Cell-cultured and X-ray-irradiation-inactivated SARS-CoV-2 virions were provided by the Virology and Pathogenesis group, UKHSA Porton Down. A dilution series of samples was produced ranging from 10^5^ to 10^1^ p.f.u. ml^−1^ (Reverse transcription - quantitative PCR(RT-qPCR) C_t_ values of 18, 25, 28, 32 and 35).

### Clinical samples

Anonymous residual Viral Transport Medium (VTM) from nasal swab samples was provided from routine UKHSA SARS-CoV-2 surveillance sequencing.

### LFD loading

Mock or clinical samples were diluted 1 : 1 v/v with the corresponding LFD sample loading buffer, and 70 µl was applied to the device via pipette. After 15 min, the LFD result was read. Ambient incubation was continued for 45 min before elution.

### Addition of preservatives

Where stated, 70 µl of the tested preservative was added to the device via the sample loading port once the result had been read. The viral sample titre used for all preservative testing was 10^3^ p.f.u. ml^−1^ (RT-qPCR C_t_ 25).

### LFD elution

#### Strip removal method

The LFD was opened manually along the join line of the outer casing, and the internal strip was removed using two pairs of tweezers, placed into a 2 ml tube containing 700 µl of MagMAX Viral/Pathogen Binding Solution (extraction buffer) and incubated for 60 min prior to nucleic acid extraction.

#### Centrifugation method

Seven hundred microlitres of extraction buffer were added to the LFD via the sample loading port. The device was placed inside a 50 ml Falcon tube, laid flat (face up) and incubated for 60 min. The tube containing the LFD was centrifuged at 2,000 r.p.m. for 2 min to elute the extraction buffer from the device into the Falcon tube prior to nucleic acid extraction.

#### Nucleic acid extraction

Samples were extracted via the KingFisher Flex platform using the MagMAX Viral/Pathogen Nucleic Acid Isolation kits and 700 µl of sample input, as per the manufacturer’s instructions.

#### SARS-CoV-2 sequencing and lineage calling

Samples were processed by the UKHSA Pathogen Genomics SARS-CoV-2 Sequencing Service, utilizing the ARTIC network tiling amplicon scheme protocol [8], with primer scheme version 5.3.2. (SARS-CoV-2 version 5.3.2 scheme release - Laboratory - ARTIC Real-time Genomic Surveillance) and sequenced on an Oxford Nanopore GridION X5. Data analysis was performed by following the ARTIC network nanopore protocol for nCoV2019 [9], and lineage was determined via Pangolin [10].

#### RT-qPCR

RT-qPCR was performed using the Luna SARS-CoV-2 RT-qPCR Multiplex Assay Kit (New England Biolabs). Assays were performed as per the manufacturer’s instructions for the multiplex detection of 2019-nCoV_N1 and 2019-nCoV_N2 targets. Reported C_t_ values are for the N1 target.

## Results

### Preliminary testing of recovery utilizing inactivated SARS-CoV-2

A titration range of inactivated SARS-CoV-2 virions was used to assess the four device brands, with genomic material recovered by removal of the test strip (see methods).

**Fig. 1.**
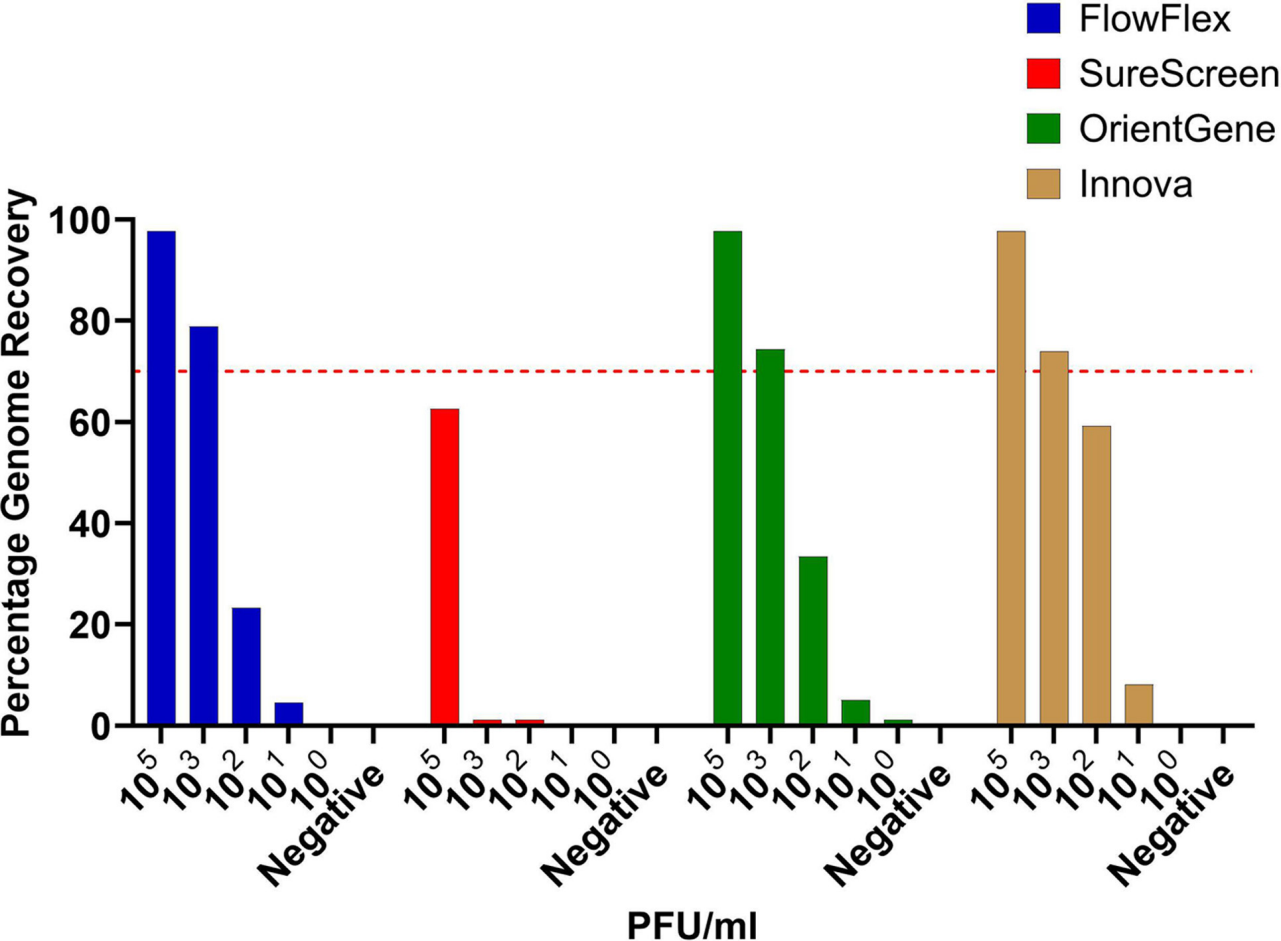
Percentage of total genome sequence recovered following elution of SARS-CoV-2 virions from four brands of LFDs. Red line indicates 70% genome recovery (approximation of level required for lineage determination).

We observed recovery of sufficient sequence information for lineage assignment (typically >70% of total genome) at both 10^5^ and 10^3^ p.f.u. ml^−1^ concentrations (equivalent to a sample C_t_ of 18 and 25, respectively) from three of the four devices tested (Fig. 1). SureScreen is the exception, with reduced genome recovery across all concentrations tested. Samples with a concentration of 10^2^ and above consistently produce a positive LFD on all brands tested.

### Clinical sample testing

To assess recoverability from a clinically relevant sample type, we tested 40 residual nasal swab VTM samples (used in COVID-19 surveillance sequencing). Residual material from each sample was loaded upon each of the four LFD types for direct comparison.

**Fig. 2.**
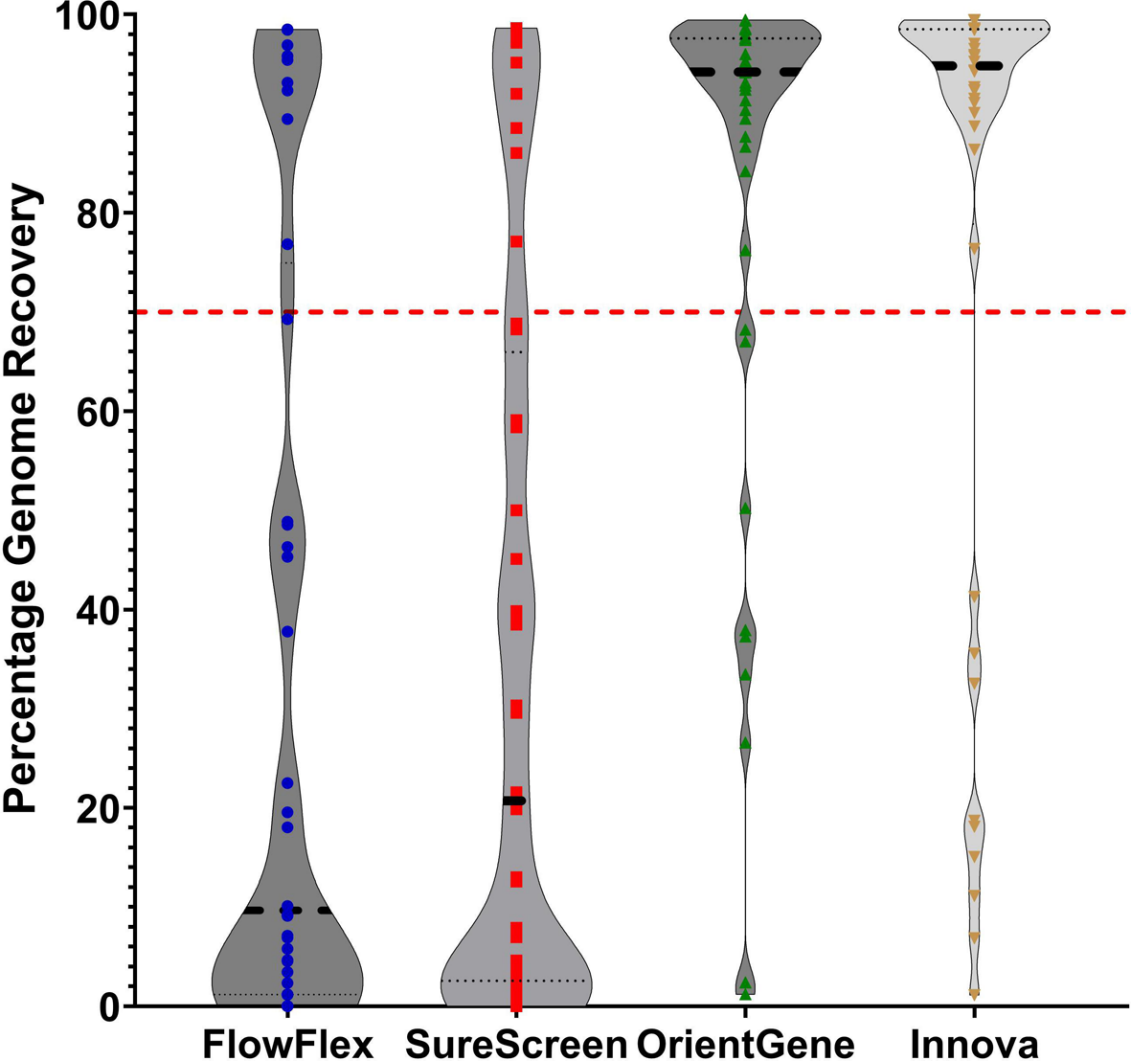
Percentage of total genome sequence recovered following elution of clinical samples across each of the four devices. Red line indicates 70% genome recovery.

Results again varied across devices. From OrientGene and Innova devices, 80% of samples achieved sufficient coverage for variant calling, whereas for FlowFlex and SureScreen devices, only 25 and 20% of samples produced sufficient coverage, respectively (Fig. 2).

In all cases where a lineage call was achieved, they were concordant with the expected result from initial sample sequencing.

### Elution efficiency and genome recovery

**Fig. 3.**
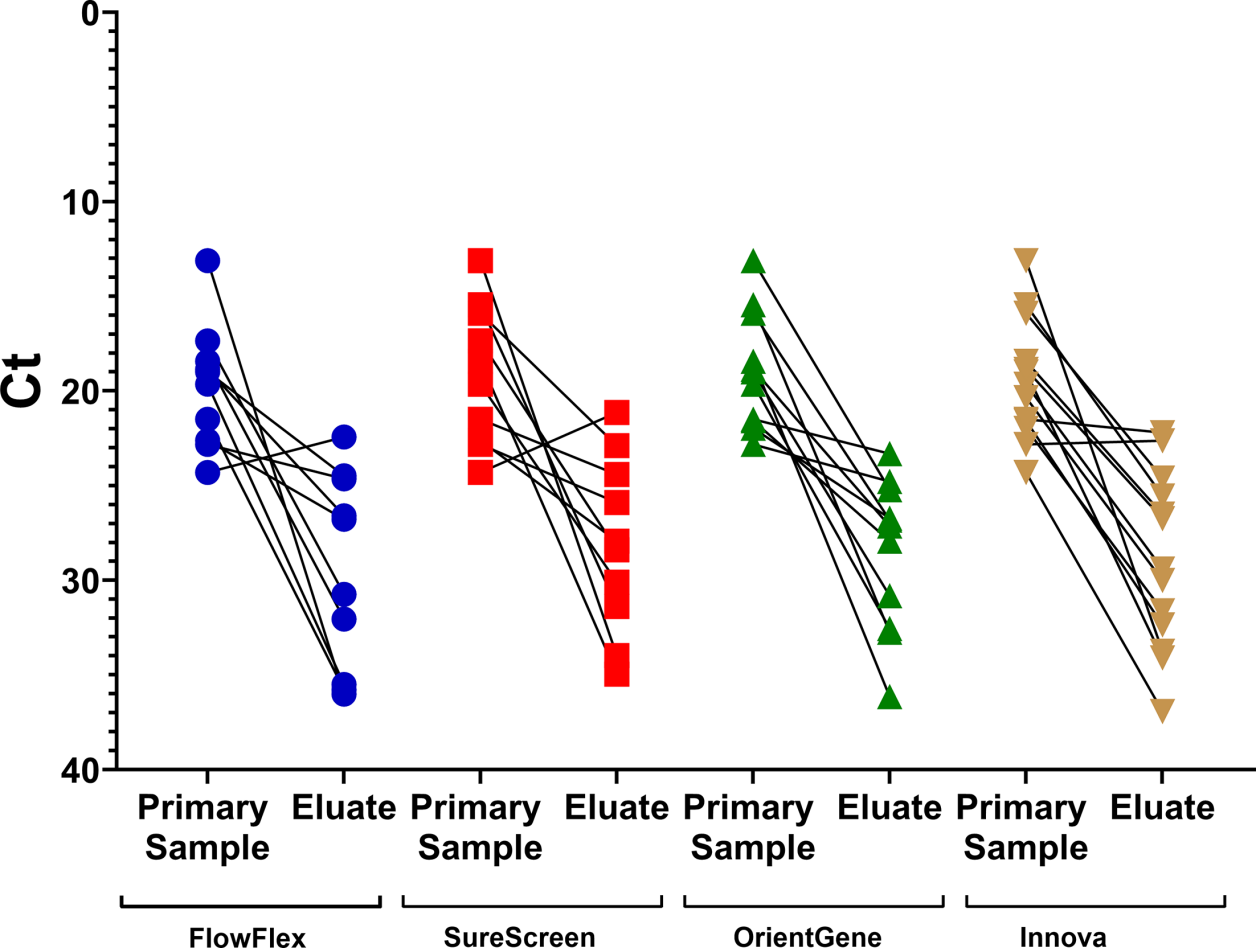
RT-qPCR C_t_ from the original sample and in the corresponding material eluted from LFD.

To assess the efficiency of elution of viral nucleic acid from the LFDs, and the effect upon sequence recovery, RT-qPCR was performed on a subset of the clinical samples and corresponding LFD eluates. A similar level of reduction in viral concentration was seen between the primary sample and the LFD eluate for all devices (Fig. 3) with mean C_t_ increases being 14.9 from FlowFlex, 13.3 from SureScreen, 11.4 from OrientGene and 12.5 from Innova.

**Fig. 4.**
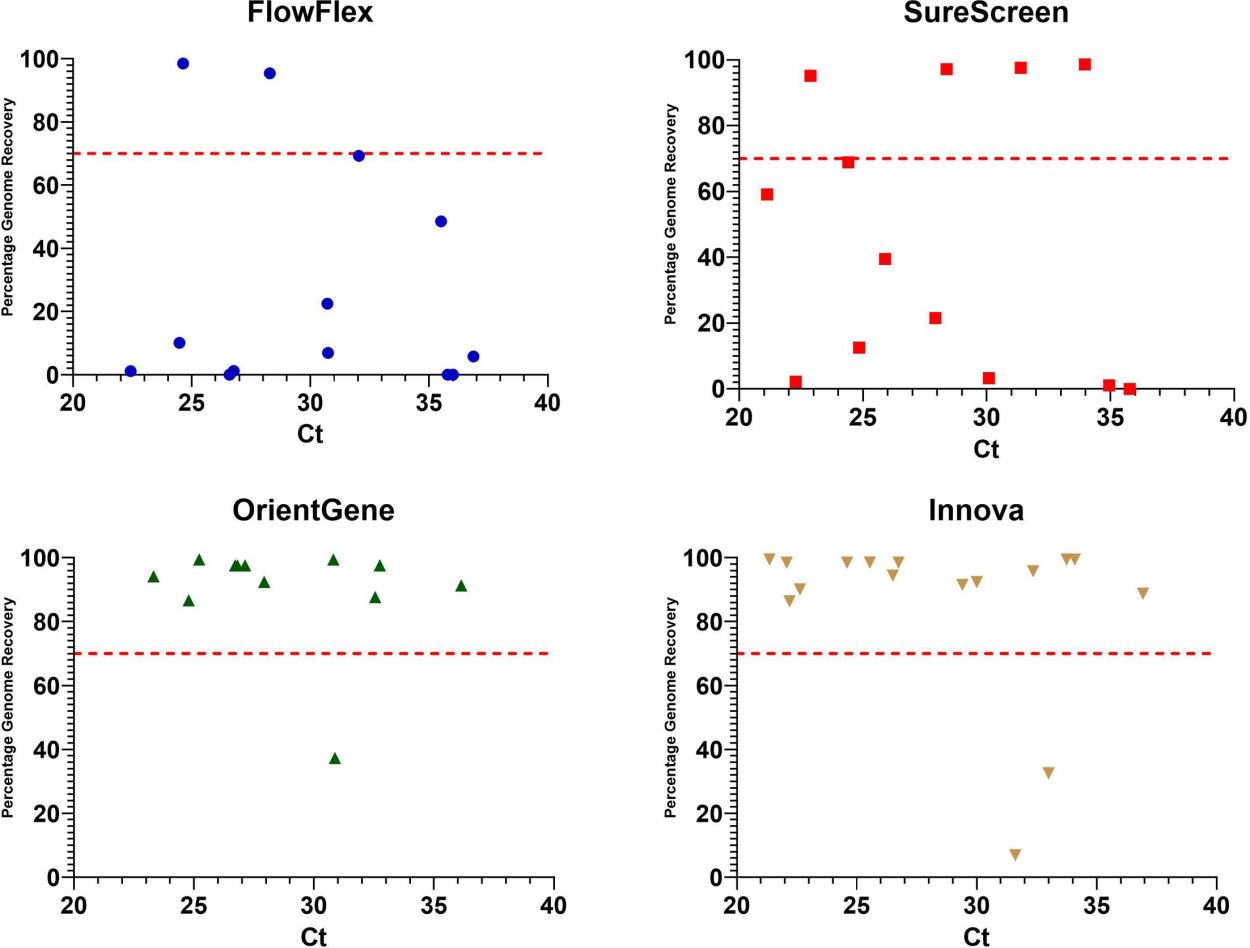
Comparison of percentage consensus genome sequence recovery from clinical samples compared to the viral titre of material eluted from the used LFDs. Red line indicates 70% genome recovery.

No clear correlation was seen between the C_t_ of the eluted material and the percentage of genome recovered for FlowFlex and SureScreen devices. For OrientGene and Innova devices, a trend was observed of C_t_ below 30 being sufficient to provide >70% genome recovery consistently, with higher C_t_ values providing more mixed results (Fig. 4).

### Process optimization and evaluation of the centrifugation method using clinical samples

Experiments initially followed the procedure for LFD strip extraction as set out by Martin *et al.* [4]. This process is time-consuming and labour-intensive; therefore, we set out to test the process of elution of sample in extraction buffer directly from the LFD via centrifugation as described by Macori *et al*. [6].

**Fig. 5.**
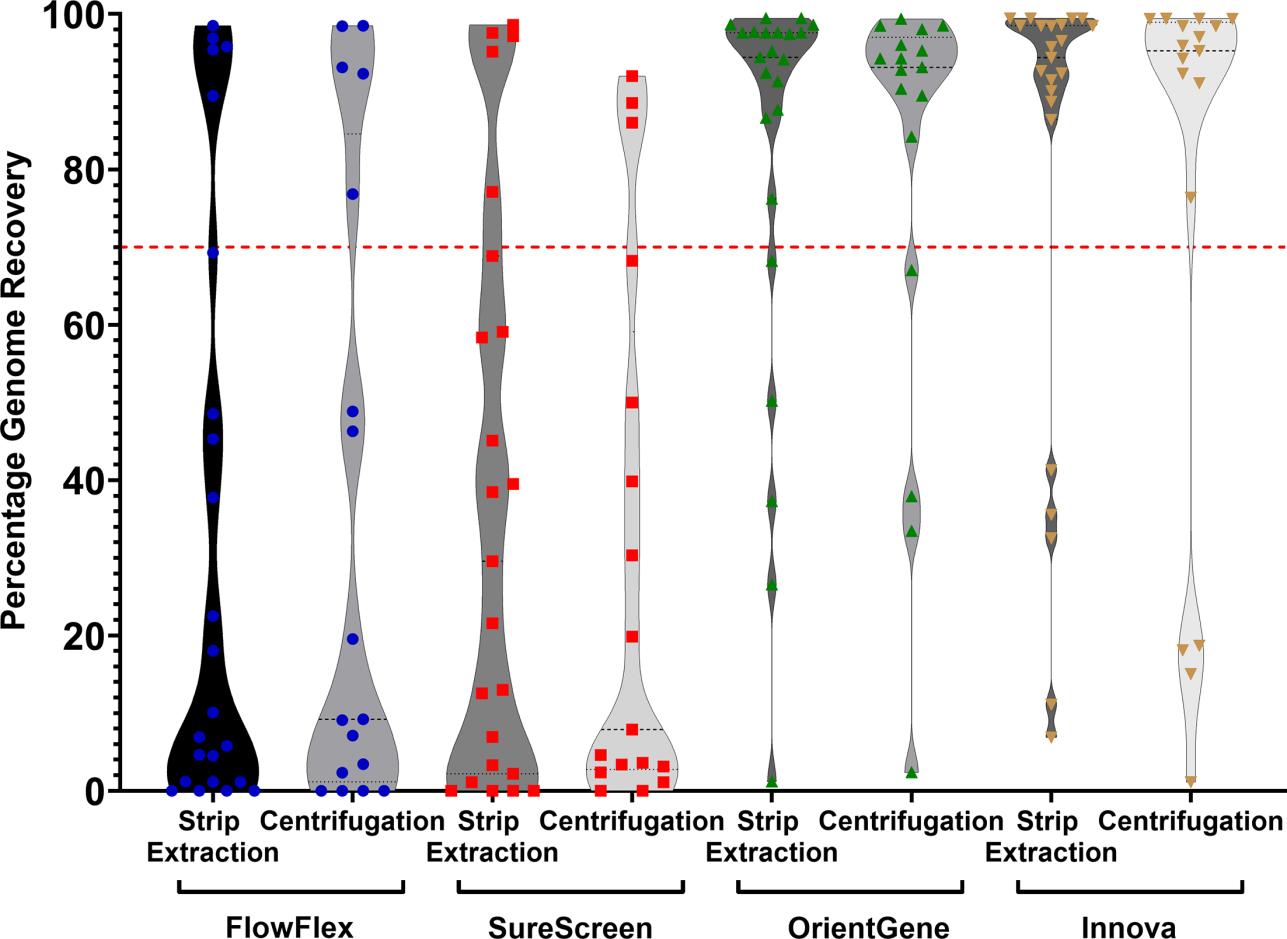
Distribution of percentage consensus genome sequence recovery following elution via strip removal (opened) or centrifugation (spun) method from clinical samples for all four LFDs tested. Red line indicates 70% genome recovery.

For initial comparison, the irradiated SARS-CoV-2 series was processed in triplicate, across three time points, on each device by both methods. LFD centrifugation provided equivalent or improved genome coverage for all device brands (see Table S1, available in the online Supplementary Material). A further comparison between the two extraction methods was made using residual clinical samples. The clinical samples tested were not paired but comprised two batches containing samples with a clinically representative C_t_ range. Results from elution by centrifugation remained comparable to those from the strip removal extraction method when using clinical samples (Fig. 5). The percentage of samples with a positive lineage call, comparing strip removal to centrifugation, was 21.7–29.4%, 21.7–17.7%, 78.2–76.4% and 78.2–76.4%, for FlowFlex, SureScreen, OrientGene and Innova devices, respectively.

### Assessment of sample viability upon LFDs over 7 days of ambient incubation

**Fig. 6.**
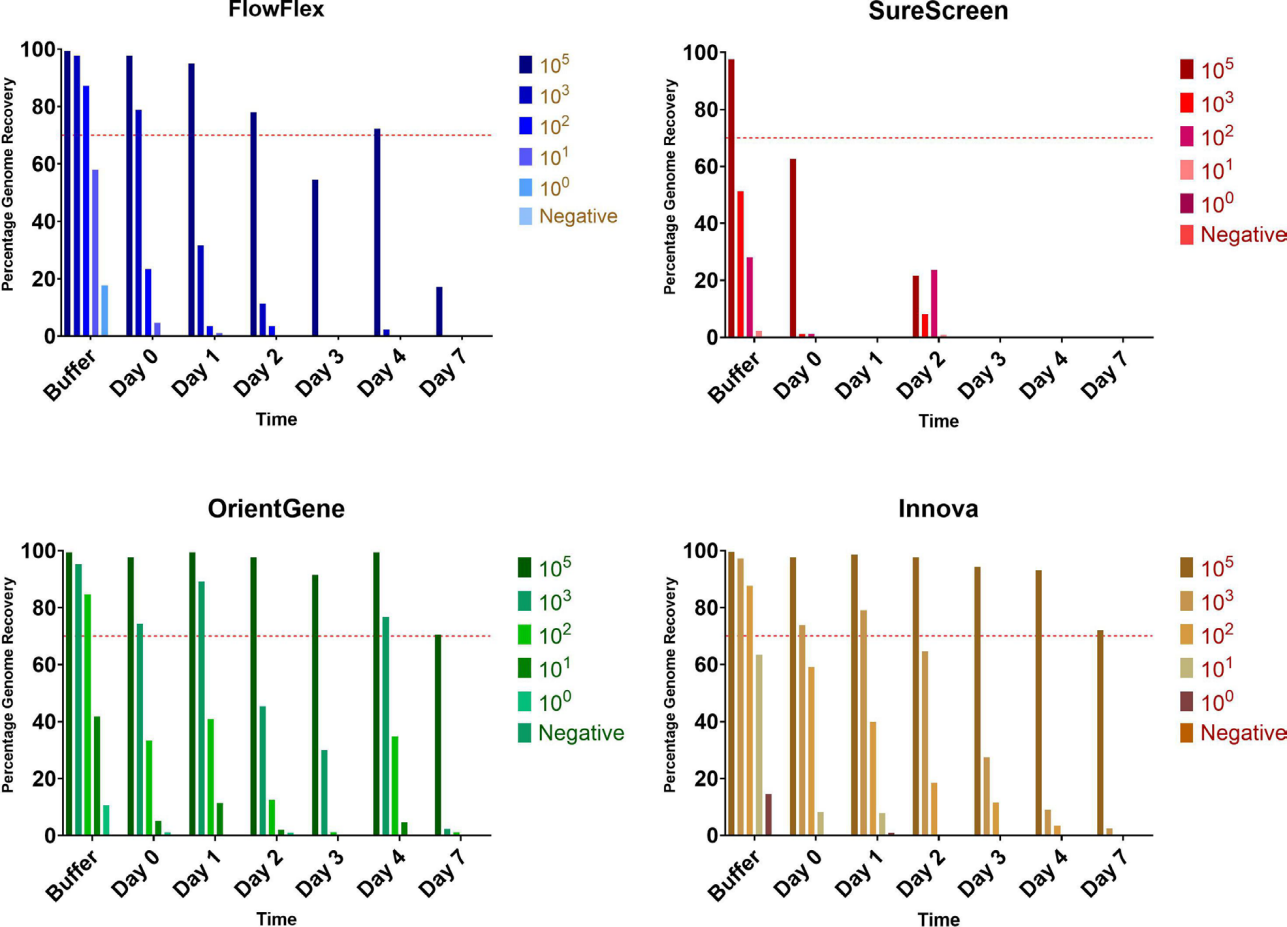
Percentage consensus genome sequence recovery following elution of a range of titres of SARS-CoV-2 virions from the four LFDs tested. Buffer indicates incubation in buffer without being run on a device. Red line indicates 70% genome recovery.

Real-world use of LFDs as a sample source for sequencing applications raises logistical issues that are likely to include delays between initial sample LFD testing and laboratory processing, particularly in home-use scenarios. We set out to assess the impact of delayed time of LFD sample receipt and processing on the ability to recover informative genome sequences. An irradiated SARS-CoV-2 dilution series was used to assess the effect of such delays ranging up to 7 days in duration. Stability of the nucleic acid in the used LFD as measured by genome sequence recovery was assessed for each device, from dilution in LFD buffer without being run on the device to running followed by multiple days of incubation upon the device post-use, at room temperature, prior to extraction (Fig. 6).

Decreased genome recovery was seen with increased delay time for all devices. The highest titre tested (equivalent C_t_ 18) provided sufficient genomic coverage for multiple days on FlowFlex, OrientGene and Innova devices (Fig. 4), but the signal rapidly degraded on SureScreen devices. The C_t_ 25 equivalent sample showed an inconsistent signal over multiple days on OrientGene and Innova devices but was rapidly degraded on FlowFlex and SureScreen devices. Decreased genome recovery was also seen for most titration series samples when extracted directly from SureScreen LFD buffer when compared to the other device buffers.

### The effect of delayed time to elution on recovery from LFDs using residual clinical sample material

Residual clinical samples were used to assess the effect of delayed time to elution on recovery from LFDs. Ten clinical samples were tested on each of 4 LFD brands over the course of 3 days. Decreased genome recovery is seen over time in all cases, but OrientGene and Innova devices still provided sufficient genome coverage for lineage calling from 60 and 50% of clinical samples tested after 2 days of incubation. FlowFlex and SureScreen produced reduced genome coverage from the samples tested, even in the absence of delayed recovery (Table 1).

**Table 1.** Percentage of clinical samples from which a sufficient genome sequence was recovered from each LFD brand following either immediate extraction for sequencing (day 0) or ambient incubation on the device (day 1 and day 2)

	Percent of samples with lineage call		
	Day 0	Day 1	Day 2
FlowFlex	30	20	0
SureScreen	30	20	0
OrientGene	90	60	60
Innova	80	70	50

### The effect of nucleic acid preservatives on recovery from LFDs over 5 days of ambient incubation

**Fig. 7.**
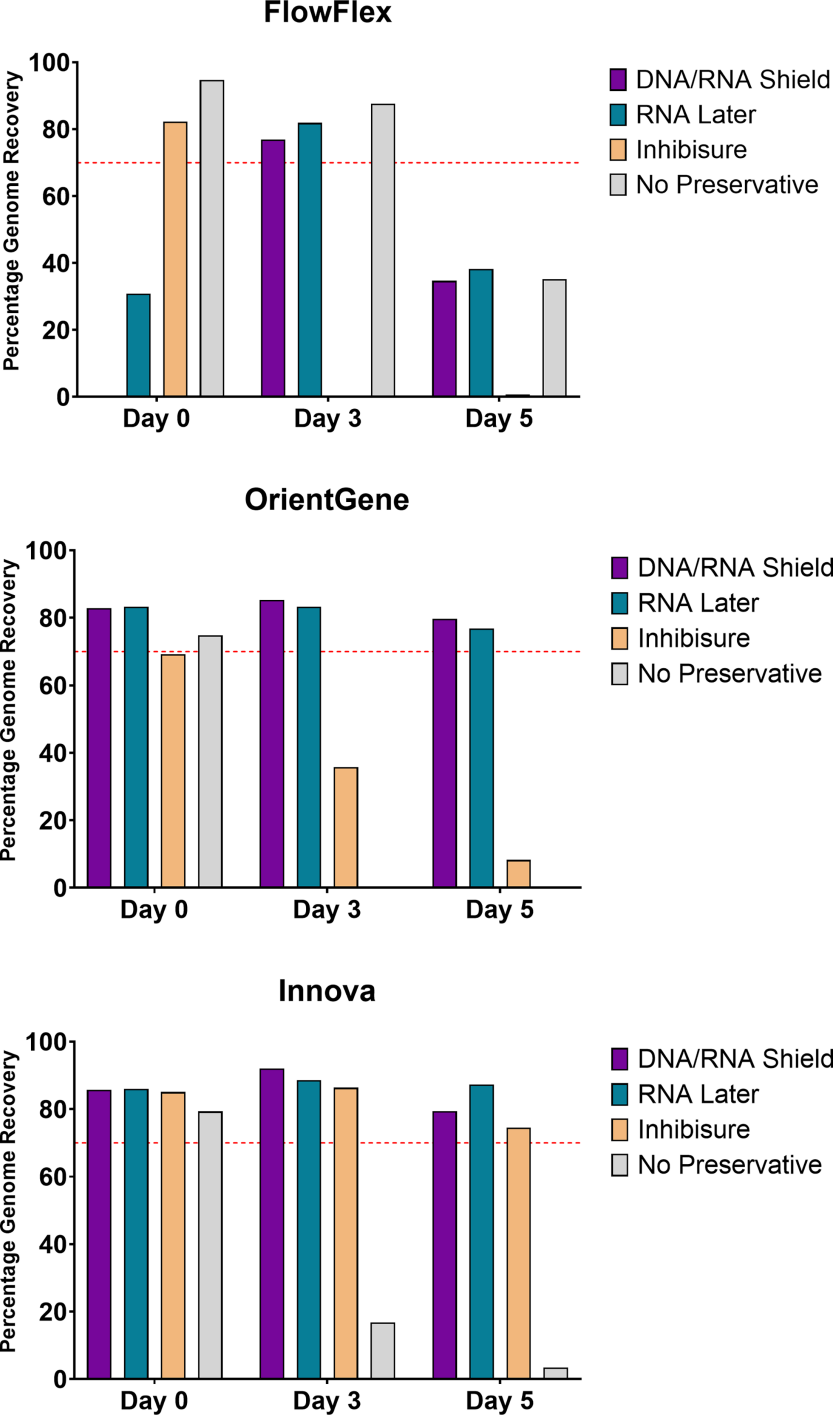
The effect of nucleic acid preservatives on genome recovery from LFDs over 5 days of ambient incubation. Red line indicates 70% genome recovery.

Addition of three RNA stabilizing agents to devices post-running of a mid-titre SARS-CoV-2 mock sample (10^3^ p.f.u. ml^−1^) shows varied results. DNA/RNA shield and RNALater preserved RNA and allowed near-complete genome sequencing after 5 days of room temperature incubation on both OrientGene and Innova devices. Inhibisure was similarly effective on the Innova device but showed no preservative effect on OrientGene devices. Results on FlowFlex were inconsistent for all preservatives tested (Fig. 7).

## Discussion

In this study, we set out to assess the feasibility of SARS-CoV-2 amplicon sequencing using LFD eluates from four major LFD brands currently in use in the UK, with the aim of generating a protocol that could subsequently be expanded to other potential use settings.

Our testing using inactivated SARS-CoV-2 observed recovery of sufficient sequence information for lineage assignment at titres as low as 10^3^ p.f.u. ml^−1^ (sample Ct ~25) from three of the four tested major devices currently in use within the UK.

Our assessment of genome recovery from 40 residual clinical nasal swab samples (C_t_ range from 13 to 24) for each LFD type found reduced genome recovery for clinical samples from FlowFlex and SureScreen devices (25 and 20% of samples with a lineage call, respectively) when compared to OrientGene and Innova devices (80 and 80% of samples with a lineage call, respectively).

Rector *et al.* [7] tested a range of LFD brands, using stored nasopharyngeal samples, and they found that LFD buffer components can be deleterious for the viral genetic material, resulting in lower RNA concentrations and interfering with sequencing. In our study, a similar reduction in viral concentration was seen in LFD eluates of all brands compared to the originating clinical samples, indicating that reduced elution efficiency is unlikely to be the cause of poorer genome recovery via amplicon sequencing in some devices. Markedly different levels of genome recovery were seen from titration series samples when extracted and sequenced directly from the buffers of the tested LFD brands. These data indicate that increased levels of degradation or the presence of inhibitors in some device buffers may be significant factors.

The process of removing strips from LFD casings for nucleic acid extraction is time-consuming, labour-intensive and not feasible for use in high-throughput processing. Work by Macori *et al.* [6] described an extraction method by centrifugation. In our study, we confirmed that LFD centrifugation provided equivalent or improved genome coverage for all device brands.

Decreased genome recovery was seen with increased delay time for all devices. OrientGene and Innova showed the best recovery over time, with 60/70% of samples showing sufficient stability after 2 days to retrieve near-complete genome sequences. For all LFD brands, considerable loss of recovery from clinically representative titrations was seen from day 3 onwards. Interestingly, Martin *et al.* [4] found that SARS-CoV-2 genomes could be successfully recovered from Abbott and InnoScreen brand test devices up to 8 days after initial sample collection.

The addition of commercial nucleic-acid-stabilizing reagents to devices after run completion showed promise. Both RNALater and DNA/RNA shield showed a preservative effect over several days upon the typically best-yielding devices, OrientGene and Innova, as has been observed by Moso *et al.* [5] for multiple RNA viruses on the PanBio COVID-19 device. Neither preservative, however, improved the recovery from the FlowFlex device.

LFDs are not designed to be compatible with extracting genetic material suitable for sequencing. However, building on the observations of Martin *et al.* [4] and others, we have verified that it is feasible to retrieve adequate genetic material for targeted amplicon sequencing from some of these devices. Our work extends this finding to various LFDs currently deployed in the UK, showing that genomic recovery varies across different devices. Accordingly, a comprehensive assessment of each LFD type is required to determine its suitability as a genomic sequencing sample source.

Consideration should be given as to whether future iterations of LFDs should incorporate the capability to efficiently sequence pathogens from the eluate as a design requirement or how this could otherwise be achieved efficiently and effectively.

We suggest the following next steps:

1. Conduct a comprehensive comparative study to quantify the efficiency of genomic recovery across different LFD brands and models working with representative suppliers. This study should aim to identify factors that influence the variability in recovery rates.
2. Engage with LFD manufacturers to share findings and collaborate on the development of next-generation devices that include the efficient recovery of genetic material as a key design objective.
3. Work towards establishing a set of guidelines or standards for evaluating LFD suitability as sources of genetic material for sequencing. These guidelines may help streamline the assessment process for new or existing LFD types.

## Supplementary material

10.1099/jmm.0.002144 Uncited Table S1.

## Abbreviations

COVID- 19: coronavirus disease 2019
LFDs: lateral flow devices
RT-qPCR: reverse transcription-quantitative polymerase chain reaction
SARS-CoV-2: severe acute respiratory syndrome coronavirus 2
UKHSA: UK Health Security Agency
VTM: viral transport medium
WGS: whole-genome sequencing
