# Supplementary material for: Assessment of SARS-CoV-2 genome sequence recovery from four lateral flow device products available in the UK
Source: J Med Microbiol. 2026 Mar 23;75(3):002144. doi: 10.1099/jmm.0.002144 (PMC13034076; doi:10.1099/jmm.0.002144)
Supplement: Uncited Table S1. [file jmm-75-02144-s001.pdf]

FlowFlex						
Viral Titre	Day 0		Day 1		Day 2	
PFU/ML	Opened	Spun	Opened	Spun	Opened	Spun
10^5	97.7	98.48	94.91	97.59	78.04	83.6
10^3	78.85	93.21	31.46	84.53	11.22	36.94
10^2	23.31	58.32	3.57	39.25	3.46	28.4
10^1	4.51	17.75	1.11	3.23	0	1.18
10^0	0	4.55	0	0	0	0

SureScreen						
Viral Titre	Day 0		Day 1		Day 2	
PFU/ML	Opened	Spun	Opened	Spun	Opened	Spun
10^5	62.62	70.04	0	7.68	21.64	26.44
10^3	1.16	19.78	0	1.19	8.13	6.79
10^2	1.19	4.51	0	0	23.57	0
10^1	0	4.48	0	0	0.85	0
10^0	0	0	0	0	0	0

OrientGene						
Viral Titre	Day 0		Day 1		Day 2	
PFU/ML	Opened	Spun	Opened	Spun	Opened	Spun
10^5	97.7	99.45	99.44	98.5	97.67	98.51
10^3	74.38	98.49	89.33	93.13	45.31	88.67
10^2	33.41	86.53	40.83	53.68	12.5	37.63
10^1	5.08	72.18	11.39	0	2.05	9.06
10^0	1.15	38.31	0	1.19	1.09	4.59

Innova						
Viral Titre	Day 0		Day 1		Day 2	
PFU/ML	Opened	Spun	Opened	Spun	Opened	Spun
10^5	97.69	99.43	98.52	99.43	97.7	92.43
10^3	73.91	97.58	79.04	97.28	64.61	82.06
10^2	59.2	94.39	39.97	86.7	18.37	49.65
10^1	8.11	75.53	7.92	29.53	0	1.09
10^0	0	30.95	0.98	2.25	0	0

Supplementary Table 1. Comparison of percentage consensus genome sequence recovery following elution via strip removal (opened) or centrifugation (spun) method of a range of titres of SARS-COV-2 virions of all four LFD devices tested. Colour coded from green (high) to red (low) coverage.
